# T cell proliferation-related genes: Predicting prognosis, identifying the cold and hot tumors, and guiding treatment in clear cell renal cell carcinoma

**DOI:** 10.3389/fgene.2022.948734

**Published:** 2022-09-02

**Authors:** Haoran Huang, Yanmin Cai, Xitao Hong, Wenzong Gao, Jun Tang, Shujuan Zhang, Zhe Xu

**Affiliations:** ^1^ Department of Pediatric Surgery, Sun Yat-sen University First Affiliated Hospital, Guangzhou, Guangdong, China; ^2^ Guangdong Provincial Key Laboratory of Diagnosis and Treatment of Major Neurological Diseases, National Key Clinical Department and Key Discipline of Neurology, Sun Yat-sen University First Affiliated Hospital, Guangzhou, Guangdong, China; ^3^ Department of Neurology, Sun Yat-sen University First Affiliated Hospital, Guangzhou, Guangdong, China; ^4^ Guangdong Provincial Key Laboratory of Organ Donation and Transplant Immunology, Guangdong Provincial International Cooperation Base of Science and Technology (Organ Transplantation), Organ Transplant Center, Sun Yat-sen University First Affiliated Hospital, Guangzhou, Guangdong, China

**Keywords:** clear cell renal cell carcinoma, T cell proliferation, prognostic model, immune, tumor microenvironment, hot and cold tumors

## Abstract

**Background:** Immunotherapy has become a new direction of current research because the effect of traditional radiotherapy and chemotherapy on clear cell renal cell carcinoma (ccRCC) is not satisfactory. T cell proliferation-related genes (TRGs) play a pivotal role in tumor progression by regulating the proliferation, activity, and function of immune cells. The purpose of our study is to construct and verify a prognostic model based on TRGs and to identify tumor subtypes that may guide treatment through comprehensive bioinformatics analyses.

**Methods:** RNA sequencing data, clinical information, and somatic mutation data of ccRCC are obtained from The Cancer Genome Atlas (TCGA) database. We identified the prognosis-related TRGs which were differentially expressed between normal and tumor tissues. After dividing the patients into a train set and a test set according to proportion 1:1 randomly, the least absolute shrinkage and selection operator (LASSO) and multivariate Cox regression analysis were performed to construct a risk-stratified model. Its prediction performance was verified. Then, Gene Set Enrichment Analysis (GSEA), principal component analysis (PCA), tumor microenvironment (TME) analysis, and the half-maximal inhibitory concentration (IC50) prediction were performed between the different groups of patients. To further discuss the immunotherapy between hot and cold tumors, we divided all patients into two clusters based on TRGs through unsupervised learning. Analyzing the gene mutation and calculating the tumor mutation burden (TMB), we further explored the relationship between somatic mutations and grouping or clustering.

**Results:** Risk-stratified model and nomogram predict the prognosis of ccRCC patients accurately. Functional enrichment analyses suggested that TRGs mainly focused on the biological pathways related to tumor progression and immune response. Different tumor microenvironment, drug resistance, and TMB can be distinguished clearly according to both risk stratification and tumor subtype clustering.

**Conclusion:** In this study, a new stratification model of ccRCC based on TRGs was established, which can accurately predict the prognosis of patients. IC50 prediction may guide the application of anti-tumor drugs. The distinction between hot and cold tumors provides a reference for clinical immunotherapy.

## Introduction

The histological subtypes of renal cell carcinoma include clear cell renal cell carcinoma (ccRCC), papillary renal cell carcinoma (pRCC), chromophobe renal cell carcinoma (chRCC), and some other rare subtypes. As the most common type of renal cell carcinoma, ccRCC accounts for more than 70% of all renal cell carcinomas. With the development of targeted therapy and immunotherapy, more and more immune checkpoint inhibitors (ICIs) have been used in clinics. For example, anti-programmed cell death protein 1(PD1) combined with anti-cytotoxic T lymphocyte antigen 4 (CTLA4) has become the first-line treatment of metastatic renal cell carcinoma. Although new targeted and immune agents continue to emerge and improve the prognosis of some patients, these drugs are still not suitable for all patients (Linehan and Ricketts, 2019; Samstein, et al., 2019; Kim, et al., 2021; Klumper, et al., 2021; Wu, et al., 2021; Wu, et al., 2022). Antineoplastic drugs are less effective in immunosuppressive tumor microenvironment (TME) (Lai, et al., 2021). The tumor immune microenvironment has become the focus of renal cell carcinoma research. Therefore, it is necessary to further study the immune landscape of ccRCC in order to promote the development of immunotherapy and improve the prognosis of patients.

Related to the proliferation and function of immune cells or tumor progression, T cell proliferation-related genes (TRGs) involve hundreds of protein-coding genes which include *CTLA4, HHLA2, PRKCQ, IL4I1, IL20RB, HOMER1, DHPS,* and so on. The ccRCC subgroup with hypomethylated CTLA4 promoter was characterized by increased infiltration of immune cells, especially CD8+T cells (Klumper, et al., 2021). IL4I1 inhibited the proliferation of T cells including CD8 + anti-tumor T cells and recruited suppressor immune cells such as Tregs by activating Aryl hydrocarbon receptor (AHR). IL4I1 promoted tumor progression by regulating TME (Lasoudris, et al., 2011; Cousin, et al., 2015; Sadik, et al., 2020). In ccRCC, HHLA2 was significantly correlated with necrosis and microvascular invasion. HHLA2/PD-L1 co-expression was significantly correlated with a high density of CD8 + and CD4 + tumor-infiltrating lymphocyte (TIL). Combined with KIR3DL3, HHLA2 inhibited T cells and NK cells. Targeting HHLA2-KIR3DL3 alone to inhibit the checkpoint pathway or in combination with PD1 blockade is a potential treatment (Zhou, et al., 2020; Bhatt, et al., 2021). After knocking down the expression of HHLA2 in human ccRCC, viability, migration, and invasion of tumor cells were significantly inhibited and the cell cycle was stagnated (Chen, et al., 2019). The function of Treg can be inhibited by PRKCQ, while PRKCQ can activate Teff (Zanin-Zhorov, et al., 2010). By inducing insulin resistance phenotype, activated PRKCQ limited the access of tumor cells to glucose. Therefore, PRKCQ has an anti-tumor effect on tumors with high glycolysis including ccRCC (Sourbier, et al., 2013). The expression of IL20RB is up-regulated in renal cell carcinoma and IL20RB had crosstalk with neutrophils (Guo, et al., 2022). *In vitro*, HOMER1 promoted the proliferation, migration, and invasion of colorectal cancer cells by up-regulating G3BP1 (Cui, et al., 2020). ERK-mediated Ser-233 phosphorylation of DHPS can affect cell proliferation, and high expression of DHPS was associated with poor prognosis of lung adenocarcinoma (Wang, et al., 2020). According to Kai-Li Liu et al., DHPS inhibitors inhibited the invasion and migration of melanoma cells (Liu, et al., 2021). TME consists of tumor cells, stromal cells, infiltrating immune cells, cytokines and other nontumour components. Positive and negative regulators of T cell proliferation, such as CTLA4, can regulate TME by affecting the clustering and number of T cells (Lai, et al., 2021). Paying attention to these regulatory genes may generate a new understanding of TME and classify tumor subtypes according to immune infiltration.

The construction of a prognostic signature has been proved to be a feasible strategy for predicting disease outcomes (Wu, et al., 2021; Wu, et al., 2022). Recently, Mateusz Legut et al. discovered some new positive regulators of T cell proliferation. It is worth noting that most of these regulators can also enhance T cell function and cytokine secretion. We found that there were 25 new TRGs (Legut, et al., 2022). Although there were many prognostic models for ccRCC patients, the prediction effect of TRGs ensemble modeling based on the fusion of 25 new genes and known TRGs is not reported. Therefore, we constructed a prognostic model based on 8 TRGs by analyzing the data of ccRCC patients in the TCGA database. Importantly, these eight genes contain the key gene CTLA4. Homer scaffold protein 1 (HOMER1), which is one of 25 newly discovered TRGs, was also contributed to the signature. What’s more, we also discussed the immune landscape and drug therapy for patients with ccRCC. The results of this study may provide alternative signature to predict the prognosis and therapeutic effect of ccRCC.

## Materials and methods

### Data and genes collected

RNA transcriptome datasets and clinical data of ccRCC patients are the latest releases from The Cancer Genome Atlas (TCGA) database (http://tcga.cancer.gov/; 29 March 2022) (Linehan and Ricketts, 2019).Somatic mutation data of ccRCC patients obtained from the TCGA database were downloaded through the University of California Santa Cruz Xena (UCSC Xena; https://xena.ucsc.edu/). (Navarro Gonzalez, et al., 2021) RNA-seq data included 541 tumor tissue samples and 72 normal tissue samples. After excluding patients with a follow-up of fewer than 30 days and missing data, we extracted clinical information from 485 patients for our survival-related study. The raw count data and TPM data from “STAR-Counts” were used for differential analysis and subsequent analyses, respectively. There were 1793 immune-related genes in the ImmPort database (https://www.immport.org/). We searched the AmiGO2 database (http://amigo.geneontology.org/amigo/) to select human protein-coding genes involved in T cell proliferation by keyword “regulation of T cell proliferation” and removed duplicates. New T cell proliferation regulators were extracted from the study performed by Mateusz Legut et al. and incorporated with the TRGs from the AmiGO2 database (Legut, et al., 2022).

### Selection of differentially expressed TRGs

Using “edgeR” and “data.table” R packages, all differentially expressed genes (DEGs) between normal and tumor tissues were selected by setting: | Log2(fold change) | > 1 and false discovery rate (FDR) <0.05. R packages “ggplot2” and “pheatmap” were used to plot volcano diagram and heatmap. Protein-protein interaction (PPI) of differentially expressed TRGs were generated through the STRING database (https://www.string-db.org/) (Szklarczyk, et al., 2021). The result was imported into Cytoscape (v3.9.0) for visualization (Shannon, et al., 2003).

### Establishment and validation of the prognostic model

Using “caret” R package, we randomly divided samples into train set and test set according to proportion 1:1. R packages “survival”, “glmnet”, and “survminer” were used for modeling and visualization. TRGs related to prognosis were screened by univariate Cox proportional hazard regression analysis. We utilized cross-validated LASSO regression to screen overall survival (OS)-related TRGs without multicollinearity. Then, a risk model based on TRGs was established by multivariate Cox regression. Each patient’s risk score can be calculated according to the model, and the formula is as follows:

$$risk\;score=\overset{n}{\underset{x=1}{\sum }}\left(coef\left(mRNAx\right)\times expr\left(mRNAx\right)\right)$$

*Coef(mRNAx)* and *expr(mRNAx)* are the survival correlation coefficient and expression of TRG involved in the construction of the model, respectively. Patients were divided into low-risk group and high-risk group according to the median risk score of all patients (Wu, et al., 2022). Univariate Cox and multivariate Cox regression analyses were performed to identify independent variables of risk score and clinical information. Besides, we visualized the accuracy of the model prediction by using “survival”, “survminer”, “pheatmap”, and “timeROC” R packages.

### Nomogram and calibration

To illustrate that the predicted results have good consistency with the actual situation, we utilized “survival”, “regplot”, and “rms” R packages to establish the nomogram and calibration curves of 1-, 2-and 3-year OS. Nomogram and calibration curves were drawn based on prognostic risk score, age, pathological grade, and tumor stage obtained from multivariate analysis.

### Function and pathway enrichment analysis

To determine the main biological properties, we use Gene Ontology (GO) to annotate the functions of TRGs, including molecular functions, cellular components, and biological pathways. Kyoto Encyclopedia of Genes and Genomes (KEGG) was used to analyze TRGs function and related high-level genome function information. We also used Gene Set Enrichment Analysis (GSEA) software (v4.2.3) to distinguish the function and pathway enrichment between high- and low-risk groups. | normalized enrichment score (NES) | > 1.5 and FDR q-value < 0.05 were considered screening conditions. “ClusterProfiler”, “org.Hs.eg.db”, “enrichplot”, “GOplot”, “ggplot2”, “grid”, “gridExtra”, and “plyr” R packages were used for visualization.

### Immune microenvironment-related research

According to the results of the GSEA analysis, we analyzed and visualized the immune microenvironment of patients in high- and low-risk groups by using “scales”, “tidyverse”, “ggpubr”, “ggExtra”, “reshape2”, “ggplot2”, “ggtext”, and “limma” R packages. Combined with the profile of infiltration estimation for all TCGA tumors downloaded from the TCGA dataset, different software including XCELL, TIMER, QUANTISEQ, MCPCOUNTER, EPIC, CIBERSORT-ABS, CIBERSORT were utilized to estimate the patients’ immune infiltration statuses. Besides, using “GSVA”, “GSEABase”, “limma”, “ggpubr”, and “reshape2” R packages, we calculated and visualized immune cell score and immune function score by single-sample gene set enrichment analysis (ssGSEA). Then, we compared the TME score and immune checkpoint activation between high- and low-risk groups by using “estimate” R package.

### Drug sensitivity

In addition, to evaluate the chemotherapeutic effect of ccRCC patients, we used “pRRophetic” R package to calculate the half-maximal inhibitory concentration (IC50) of chemotherapeutic drugs. The result may guide individualized treatment.

### Somatic mutation analysis and tumor mutation burden

The somatic mutations of TRGs involved in constructing the prognostic model were obtained from the cBioPortal database (https://www.cbioportal.org/). According to the “VarScan2 Variant Aggregation and Masking” data downloaded through UCSC Xena, the differentially expressed TRGs mutations of patients in high- and low-risk groups were analyzed and visualized by using “GenVisR” R package. Representing the number of mutations per million bases in tumor tissue, TMB was associated with the prognosis of patients (Samstein, et al., 2019). We showed the mutation landscape of ccRCC patients in the TCGA database and calculated the TMB score for each patient by using “maftools”, “AnnotationDbi”, “SummarizedExperiment”, “tidyverse”, “TCGAbiolinks”, and “org.Hs.eg.db”R packages. Then we assessed the correlation between TMB score and risk score based on the stratified model.

### Clusters based on 8 prognostic TRGs

To explore the potential molecular subsets, we used the “ConensusClusterPlus”, “Rtsne”, and “scatterplot3d” R packages to identify the subgroups and performed 3D principal component analysis (PCA), t-distributed stochastic neighbor embedding (t-SNE), and Kaplan-Meier survival analysis. We also performed immune microenvironment-related analysis, calculated the TMB scores, and investigated the drug sensitivities between different clusters to explore the similarities and differences between clusters and high- and low-risk groups.

### Statistical analysis

All statistical analyses were carried out in R language (v4.1.3). Single-factor analysis of variance was utilized to compare gene expression between normal tissues and tumor tissues of ccRCC, and FDR was calculated by Benjamini–Hochberg method. Univariate Cox, cross-validated LASSO, and multivariate Cox regression analyses were used to screen overall survival (OS)-related TRGs which contributed to the risk model. The overall survival rates of different groups and clusters were estimated by Kaplan-Meier method, and the significance was tested by log-rank. The independent prognostic value of the risk signature regarding OS was evaluated by univariate and multivariate Cox regression analyses. Wilcoxon rank-sum test was used for the inter-group comparisons. Spearman rank correlation was used to calculate the correlations between TMB and risk scores, between TMB and expression of TRGs, and between immune cells infiltration scores and risk scores, respectively. All statistical tests take *p* < 0.05 as statistically significant.

## Results

### Identification and analyses of differentially expressed TRGs

**FIGURE 1:**
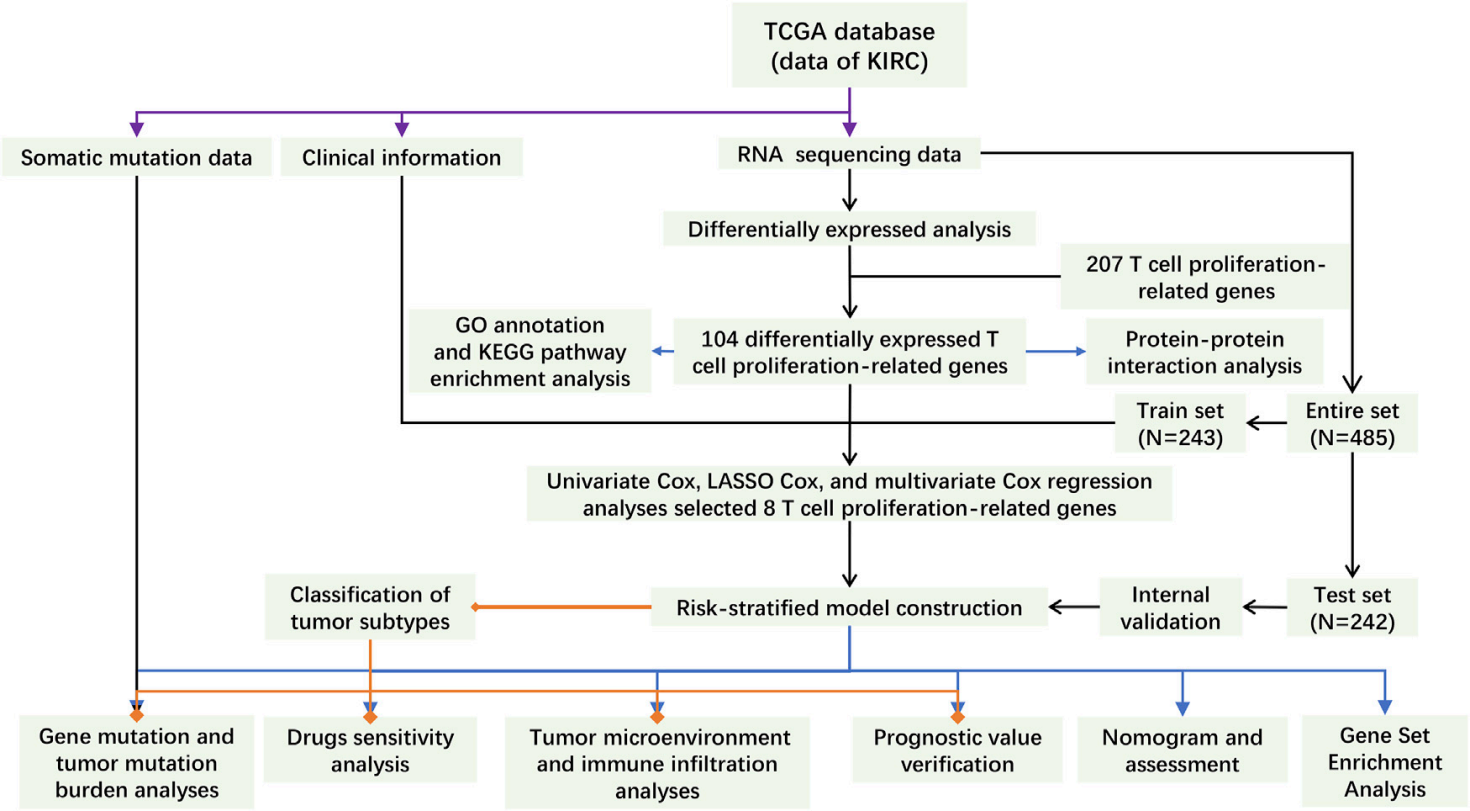
The flowchart of this study.

**FIGURE 2:**
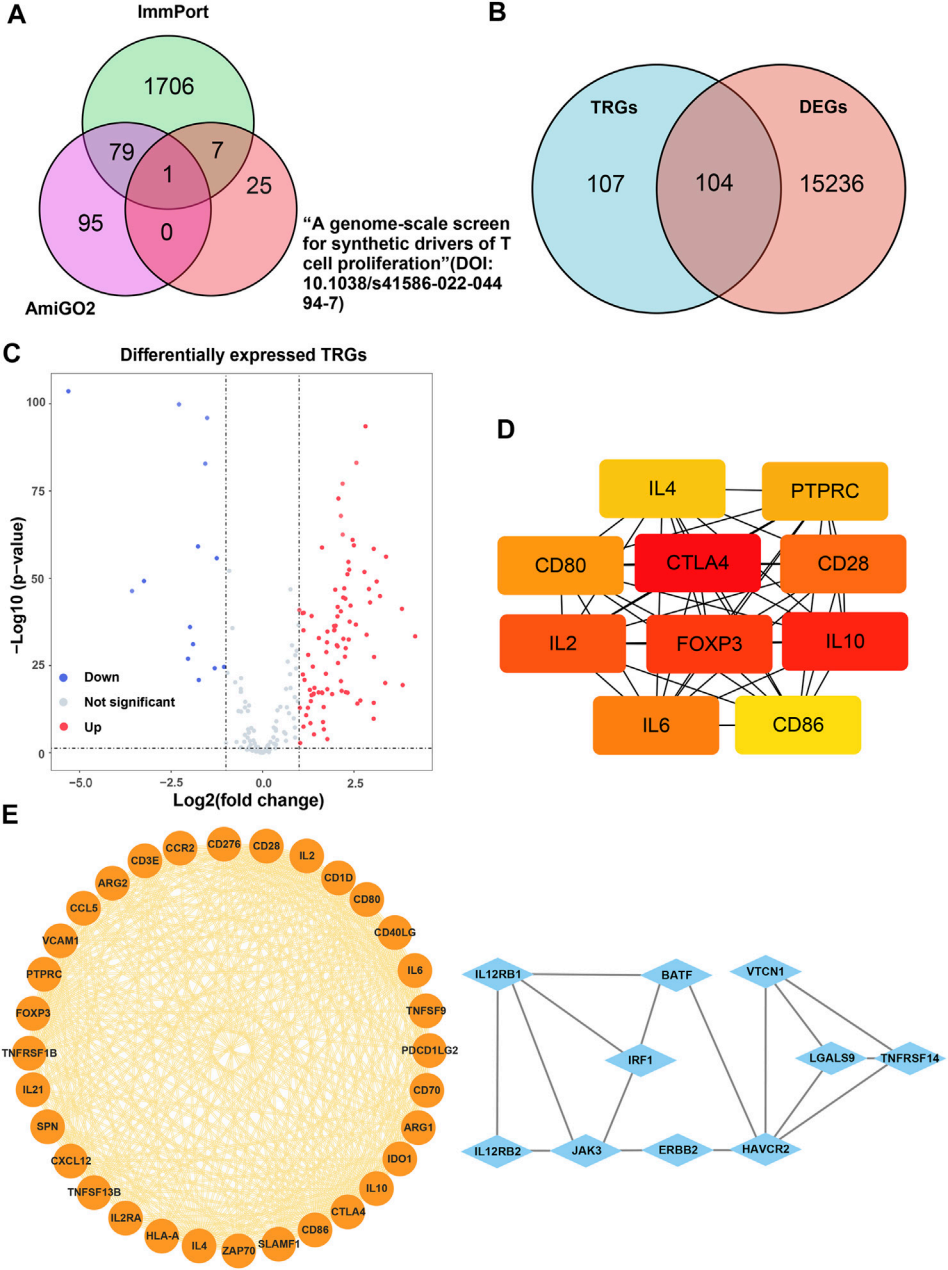
Analysis of the differentially expressed TRGs. **(A)** The Venn diagram depicting intersecting genes in the newly found TRGs and different databases; **(B)** the Venn diagram depicting intersecting genes in TRGs and DEGs; **(C)** the volcano plot of differentially expressed TRGs; **(D)** the interconnection of 10 hub differentially expressed TRGs, darker color represented higher scores; **(E)** the visualized PPI network of differentially expressed TRGs obtained by using “MCODE” plugin of Cytoscape.

The main process of this study is shown in Figure 1. A total of 207 TRGs with protein-coding functions were obtained from databases and an article (Legut, et al., 2022) (Figure 2A; Supplementary Table S1). A total of 104 differentially expressed TRGs were obtained by the intersection of all DEGs with the TRGs gene set. Ninety up-regulated TRGs and fourteen down-regulated TRGs were found in the differential analysis between ccRCC and normal kidney tissues (Figure 2B; Supplementary Table S2). We drew a volcano diagram to show the differentially expressed TRGs (Figure 2C). Using the analysis function of the STRING database, we constructed a PPI network of differentially expressed TRGs. The top 10 hub genes and pivotal modules were obtained through the “cytohubba” plugin and “MCODE” plugin of Cytoscape, respectively (Figures 2D,E). We found that the top 10 hub genes included CTLA4, FOXP3, CD28, CD80, CD86, and IL2/4/6/10. According to the previous study, CD28 can activate the PI3K/Akt/mTOR pathway, which is closely related to the promotion of T cell growth and proliferation. CTLA-4 and PD-1 can affect T cell proliferation and function by inhibiting signal molecules in this pathway (Maciver, et al., 2013).

### Construction, validation, and evaluation of the model

**FIGURE 3:**
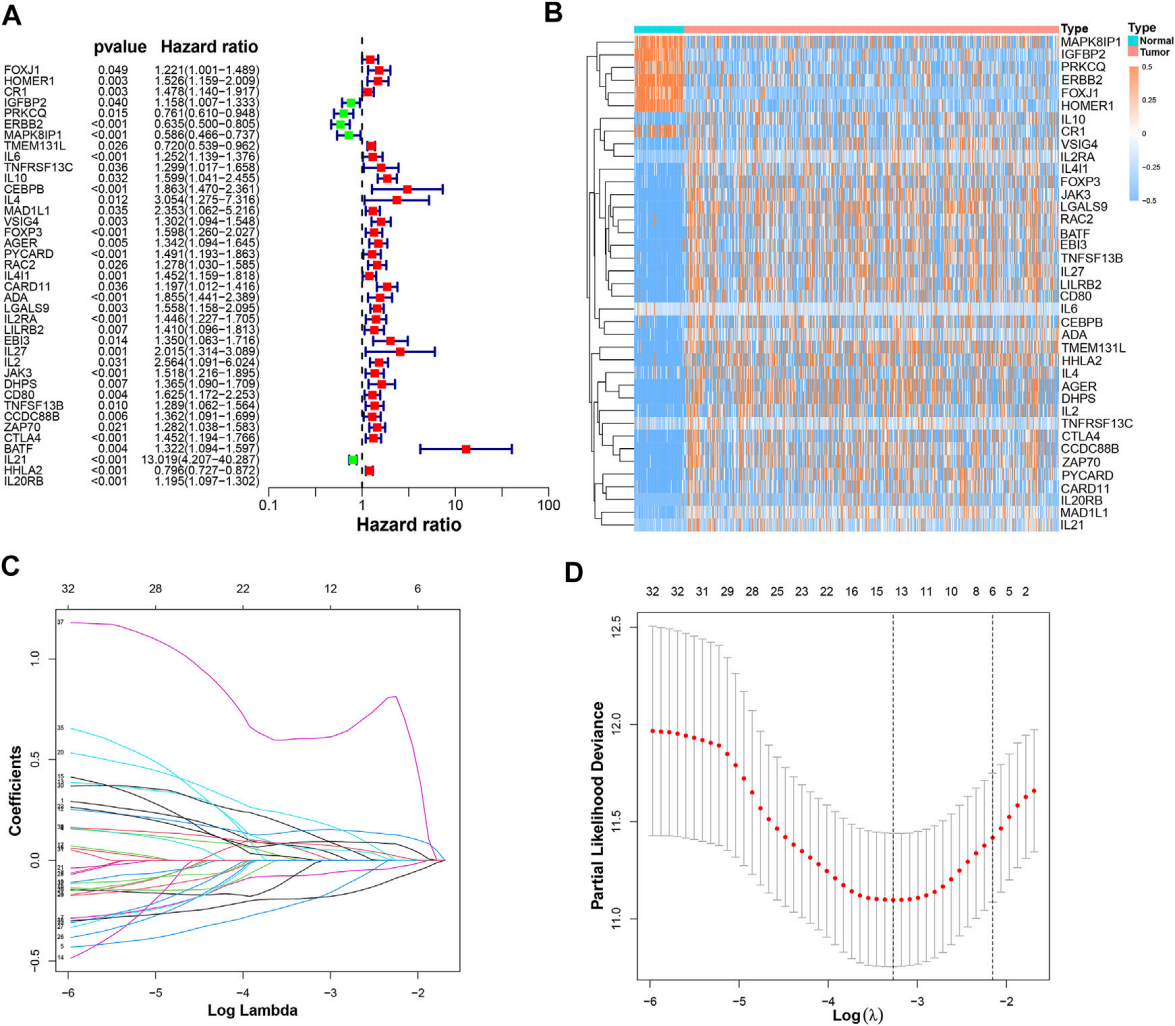
The construction of a prognostic model. **(A)** 39 prognostic TRGs extracted by univariate Cox regression analysis from 104 differentially expressed TRGs; **(B)** the heatmap of these 39 prognostic TRGs; **(C)** the LASSO coefficient profiles of these 39 prognostic TRGs; **(D)** the 10-fold cross-validation for variable selection in the LASSO model.

It was found that 39 mRNAs related to T cell proliferation were significantly correlated with OS through univariate Cox regression analysis (Figure 3A). We drew a heatmap based on the expression of 39 TRGs (Figure 3B). Performing LASSO regression, 14 TRGs were extracted when the first-rank value of Log(*λ*) was the minimum possibility of deviation (Figures 3C,D). Then we performed multivariate Cox regression analysis and got 8 TRGs to construct a risk-stratified model.

The risk scores were calculated as follows: risk score = CTLA4 × (0.2337) + HOMER1 × (0.2690) + Protein kinase C theta (PRKCQ) × (−0.2833) + Transmembrane 131 like (TMEM131L) × (−0.3749) + Interleukin 4 induced 1(IL4I1) × (0.4199) + Deoxyhypusine synthase (DHPS) × (0.3211) + HERV-H LTR-associating 2 (HHLA2) × (−0.2633) + Interleukin 20 receptor subunit beta (IL20RB) × (0.1564).

**FIGURE 4:**
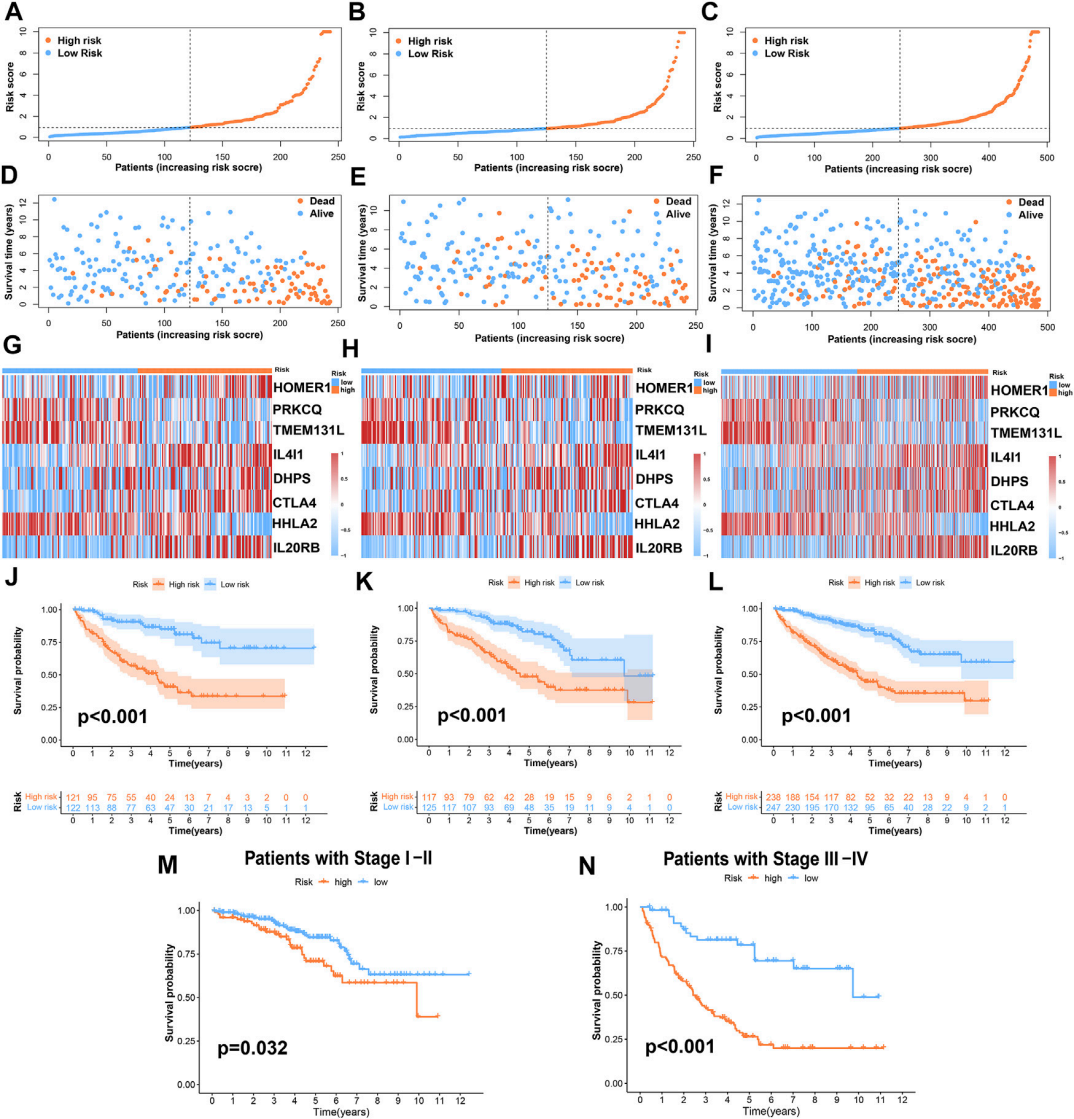
Prognosis value of the risk-stratified model in the train, test, and entire sets. **(A–C)** The risk-stratified model was based on 8 TRGs of the train, test, and entire sets, respectively; **(D–F)** the exhibition of survival time and survival status between low- and high-risk groups in the train, test, and entire sets, respectively; **(G–I)** the heatmap of 8 TRGs in the train, test, and entire sets, respectively; **(J–L)** Kaplan–Meier survival curves of OS of patients between low- and high-risk groups in the train, test, and entire sets, respectively. **(M,N)** Kaplan–Meier survival curves of OS of patients between low- and high-risk groups stratified by tumor stage.

According to the risk score formula, we divided the patients into high- and low-risk groups on average. To evaluate the difference in survival time and survival state between the two groups of patients, we drew survival curves, heatmaps, and so on (Figures 4A–L). As can be seen from the figures, the prognosis of the high-risk group was significantly worse than that of the low-risk group, and there was a significant difference in the expression of 8 TRGs participating in the building model between these two groups. Importantly, the model is suitable not only for patients with early tumor staging but also for patients with advanced stages (Figures 4M,N).

**FIGURE 5:**
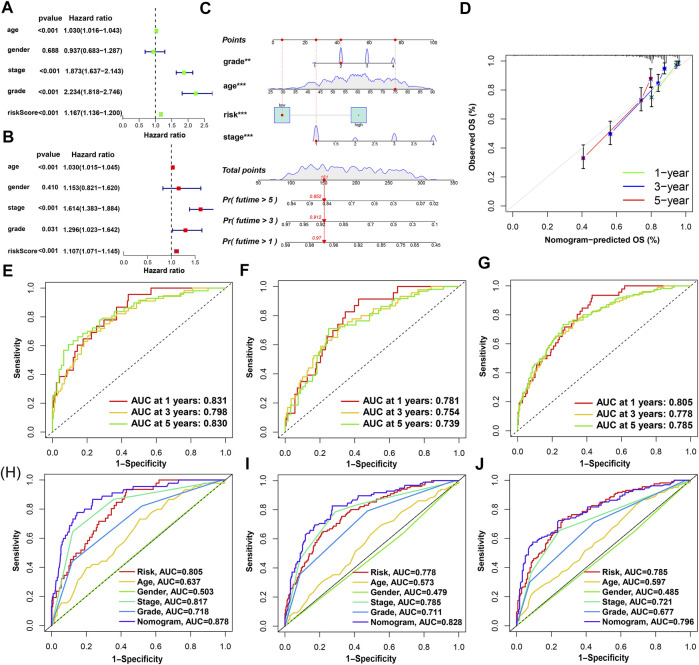
Nomogram and assessment of the risk-stratified model. **(A,B)** Univariate Cox and multivariate Cox regression analyses of clinical factors and risk score with OS, respectively; **(C)** the probability of the 1-, 3-, and 5-year OS predicted by the nomogram which integrated the risk score, age, tumor grade, and tumor stage; **(D)** the calibration curves for 1-, 3-, and 5-year OS; **(E–G)** the 1-, 3-, and 5-year ROC curves of the train, test, and entire sets, respectively; **(H–J)** the 1-, 3-, and 5-year ROC curves of risk score, nomogram score, and clinical characteristics.

The results of univariate Cox and multivariate Cox regression analysis of clinical information were consistent (Figures 5A,B). There was no significant correlation between gender and prognosis, while age, pathological grade, tumor stage, and risk score were negatively correlated with good prognosis. Among the univariate Cox analysis results, the hazard ratios (HR) and 95% confidence interval (CI) of the risk score were 1.167 and 1.136–1.200 (*p* < 0.001), respectively. In multivariate Cox regression analysis, the HRs of risk score, age, tumor stage, and pathological grade were 1.107, 1.030, 1.614, and 1.296, respectively (*p* < 0.05). As independent prognostic factors, risk score, age, tumor stage, and pathological grade were used to create nomogram plots that predicted 1-, 3-, and 5-year OS (Figure 5C). In addition, the 1-, 2-, and 3-year calibration plots proved that the OS predicted by the nomogram was consistent with actual conditions (Figure 5D). We plotted 1-, 3-, and 5-year time-dependent receiver operating characteristics ROC curves to assess the sensitivity and specificity of the prognosis of our model (Figures 5E–G). The area under the ROC curve (AUC) of the risk score in the training group was as high as 0.831, and the AUC values of the test group were also greater than 0.7. The AUC values of the entire set for 1-, 3-, and 5-year were 0.805, 0.778, and 0.785, respectively. This shows that the prediction accuracy of our model is relatively high. In addition, ROC curves for risk score, clinical information, and nomogram score were plotted (Figures 5H–J). The 1-, 3-, and 5-year AUC values of the nomogram score were 0.878, 0.828, and 0.796, respectively, which showed high accuracy.

### Function and pathway enrichment analysis

**FIGURE 6:**
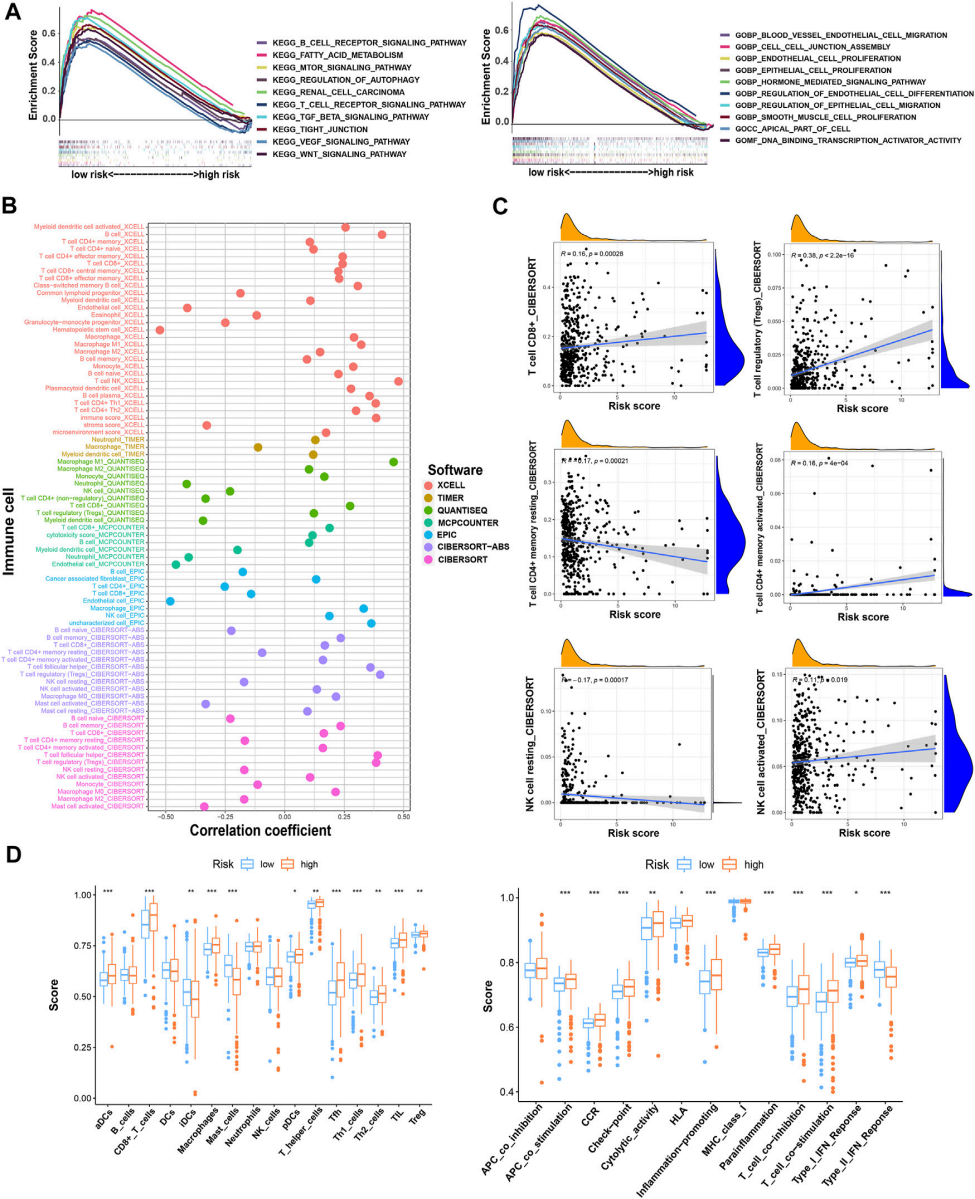
The investigation of function and pathway enrichment and tumor immune microenvironment between the high- and low-risk groups. **(A)** GSEA of the top 10 functions and pathways significantly enriched in the low-risk group; **(B)** the immune cell bubble of risk groups; **(C)** the correlation between risk score and some of the tumor immune cells; **(D)** the comparison of ssGSEA score including immune cell score and immune-related function score between risk groups. * means *p* < 0.05; ** means *p* < 0.01; *** means *p* < 0.001.

We analyzed the pathway and function enrichment of differentially expressed TRGs by KEGG and GO, which suggested that it was mainly enriched in immune-related pathways and T cell proliferation and activation (Supplementary Figure S1). Using GSEA software, we analyzed the pathway and function by “c2.cp.kegg.v7.5.1.symbols.gmt” and “c5.go.v7.5.1.symbols.gmt” of gene sets database in patients with high- and low-risk groups. Interestingly, GSEA enrichment was mainly concentrated in the low-risk group, while the FDR values of the high-risk group were all greater than 0.25. Therefore, we selected the results of interest in the low-risk group to display (*p* < 0.05; FDR < 0.05; |NES| > 1.5). Compared with the high-risk group, the low-risk group mainly enriched tumor-related and metabolic-related pathways and functions (Figure 6A).

### Estimation of intratumoral immune cell infiltration

**FIGURE 7:**
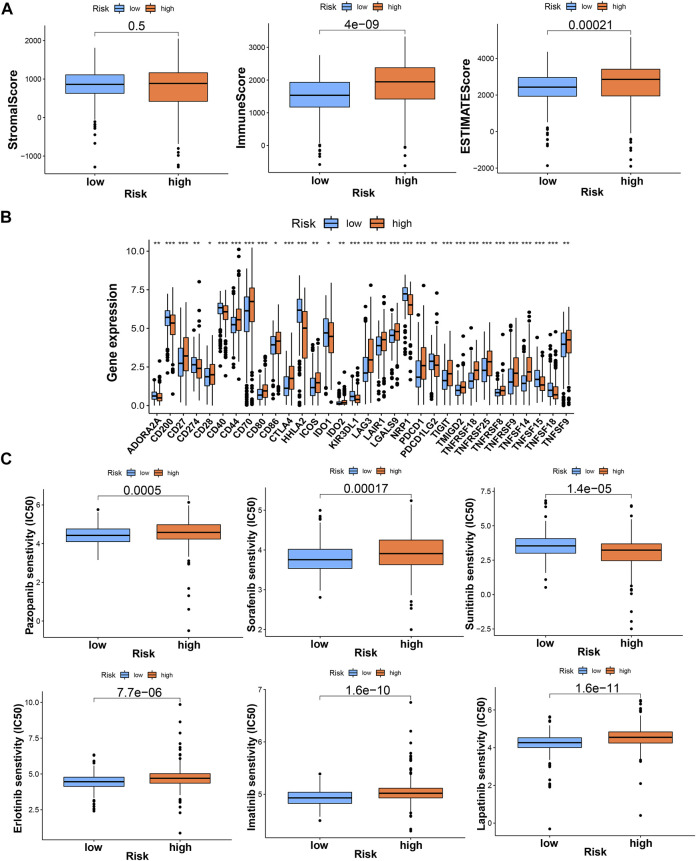
The investigation of tumor immune microenvironment and drug sensitivity between the high- and low-risk groups. **(A)** The comparison of the stromal score, immune score, and estimate score between risk groups; **(B)** the difference of checkpoints expression between risk groups; **(C)** some of the drug sensitivity predictions of risk groups. * means *p* < 0.05; ** means *p* < 0.01; *** means *p* < 0.001.

By using different software for immune cell correlation analysis, we found that immune score, microenvironment score, and cytotoxicity score have a stronger correlation in the high-risk group than the low-risk group (Figure 6B; Supplementary Table S3). The high-risk group had more types of immune-associated cells than the low-risk group. For example, Macrophage M0, Macrophage M1, plasmacytoid dendritic cell, and cancer-associated fibroblast are positively correlated with the risk score. However, eosinophil and endothelial cells were negatively correlated with the risk score (*p* < 0.05). Interestingly, we found that resting CD4^+^ memory T cells and resting NK cells were more correlated with low-risk scores. However, activated NK cells and activated CD4^+^ memory T cells were more closely associated with high-risk scores. Importantly, CD8+T cells and Tregs were strongly associated with high-risk scores (Figure 6C). Therefore, we speculated that the high-risk patients may have a higher state of immune cell infiltration. Boxplots were created to show differences in immune cells, immune-related functions, and TME in the high- and low-risk groups (Figure 6D). We calculated the TME scores of patients (Supplementary Table S4). Although there was no significant difference in stromal score in high- and low-risk groups, immune cell score and estimate score were different significantly (*p* < 0.05) (Figure 7A). Given the differences in immune cell correlations, we also analyzed immune checkpoints in these two groups. The results indicated that there were significant differences at 32 immune checkpoints in the high-low risk group, with 23 of them with *p* < 0.001 (Figure 7B). This suggested that we can group ccRCC patients and select appropriate checkpoint inhibitors.

**FIGURE 8:**
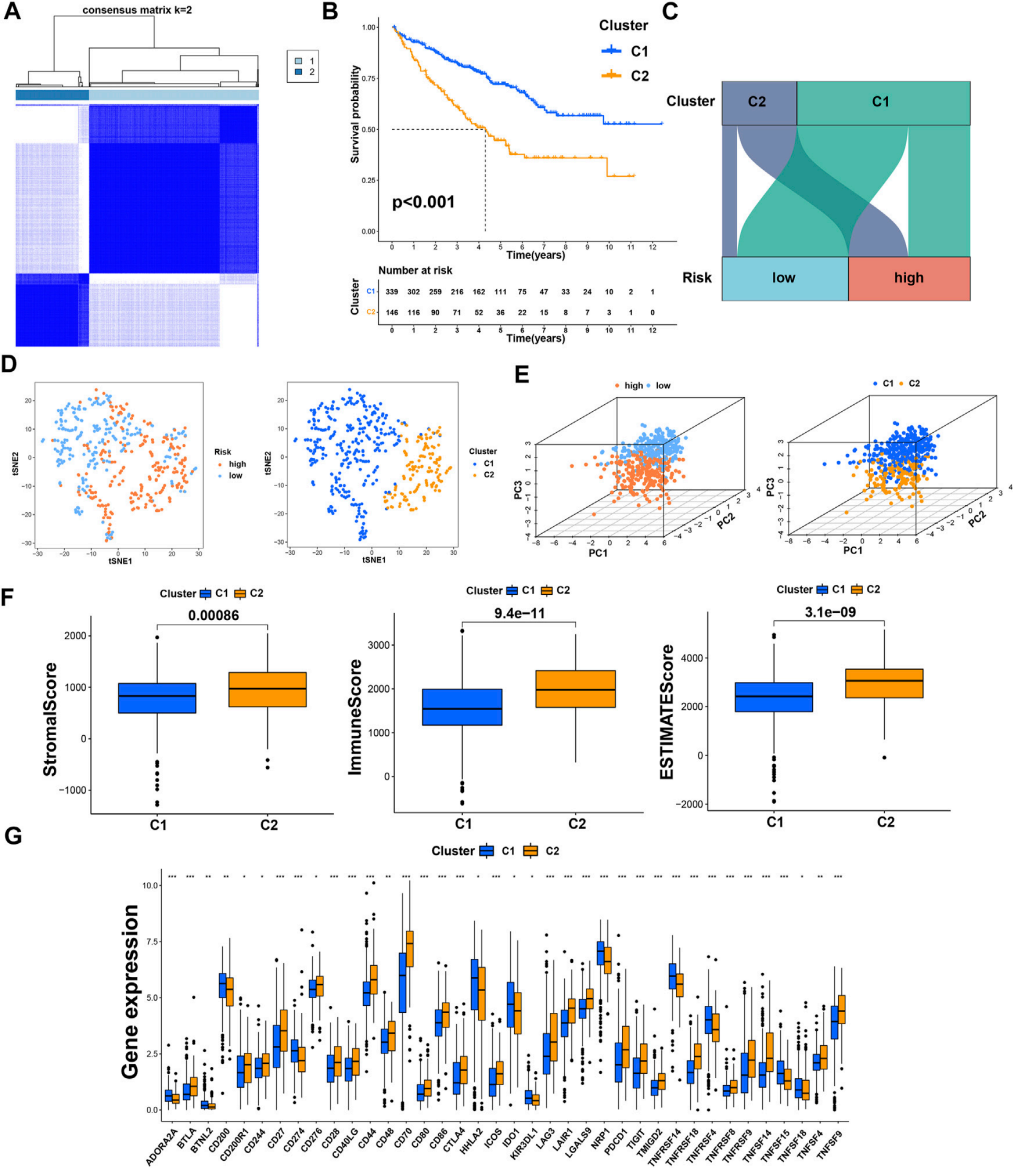
Distinction between risk groups and clusters. **(A)** Patients were divided into two clusters according to tumor subtypes; **(B)** Kaplan–Meier survival curves of OS in clusters; **(C)** the Sankey diagram of risk groups and clusters; **(D)** the t-SNE of risk groups and clusters; **(E)** the 3D PCA of risk groups and clusters; **(F)** the comparison of the stromal score, immune score, and estimate score between clusters; **(G)** the difference of checkpoints expression between clusters. * means *p* < 0.05; ** means *p* < 0.01; *** means *p* < 0.001.

**FIGURE 9:**
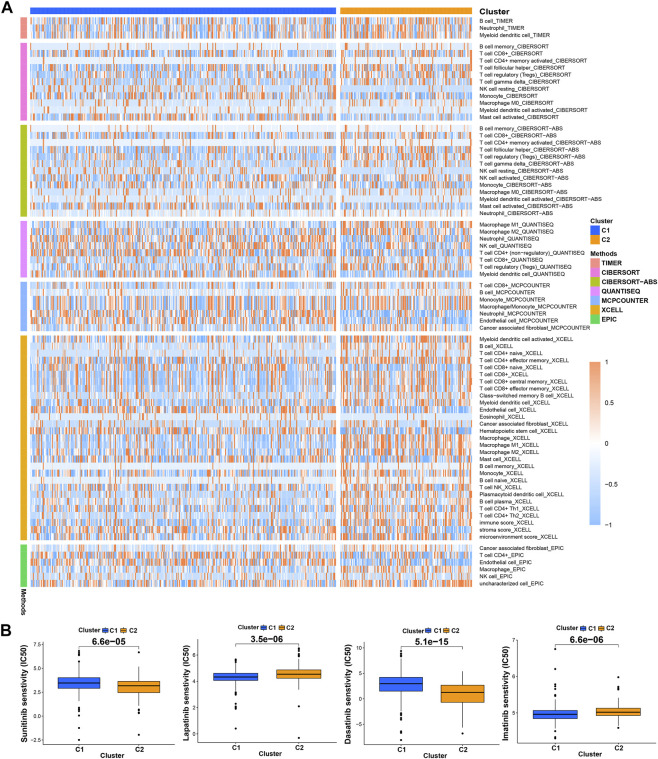
Distinction between risk groups and clusters. **(A)** The heatmap of immune cells in clusters from different platforms; **(B)** some of the drug sensitivity prediction of clusters.

Previous studies have proved that tumors divided into different subtypes often have different immune microenvironments and respond differently to immunotherapy. For the subtypes of ccRCC, the increased infiltration of immune cells suggests that these tumors are immune “hot tumors”, otherwise they are called “cold tumors” (Galon and Bruni, 2019; Klumper, et al., 2021). To distinguish cold and hot tumors in ccRCC, patients were regrouped into two clusters by R package “ConensusClusterPlus” based on the expression levels of the 8 TRGs involved in modeling (Figure 8A; Supplementary Table S6). For different clusters, the curves in the Kaplan-Meier analysis showed significant differences (*p* < 0.001) (Figure 8B). To compare the similarities and differences between clusters and risk groups, we drew the Sankey diagram and performed PCA and t-SNE. Cluster1 had a better prognosis, while Cluster2 had a poor prognosis. Patients in Cluster1 mostly belong to the low-risk group, while patients in Cluster2 were mostly part of the high-risk group. We can clearly distinguish these two clusters by t-SNE. The differences between clusters can be seen more clearly through 3-dimensional PCA than 2-dimensional PCA (Figures 8C–E). Patients with subtypes were able to distinguish TME significantly. Cluster1 and cluster2 had significant differences in the stromal score, immune score, and estimate score (*p* < 0.001) (Figure 8F). In the analysis of 47 immune checkpoints, 38 checkpoints showed heterogeneity between different clusters (*p* < 0.05). Importantly, the *p* values of 27 checkpoints were less than 0.001 (Figure 8G). The score on immunity and microenvironment of cluster2 was higher than that of cluster1. Analysis of immune cell infiltration by different software showed that neutrophil, endothelial cell, B cell, monocyte, fibroblast associated with cancer, myeloid dendritic cell, NK cell, and T cell were significantly different between different clusters (*p* < 0.05) (Figure 9A; Supplementary Table S7).

### Drug sensitivity

Using “pRRophetic” R package, we screened potential therapeutic drugs. The results suggested that the high-risk group had a lower IC50 value (indicating higher sensitivity) in 33 targeted agents (e.g., A.443654) and a higher IC50 value in 26 targeted agents (e.g., AS601245) (*p* < 0.05) (Figure 7C; Supplementary Table S5; Supplementary Figure S2). As for the first-line agents of ccRCC, patients in the high-risk group were sensitive to Sunitinib, while patients in the low-risk group were sensitive to Pazopanib and Sorafenib. Unlike the risk grouping, it was found that 56 targeted agents such as Sunitinib had significant differences between these two clusters (*p* < 0.05). Interestingly, 42 targeted agents had lower IC50 in Cluster2, while there were only 14 targeted agents had lower IC50 in Cluster1 (Figure 9B; Supplementary Table S8; Supplementary Figure S3). For clusters based on 8 TRGs, precise drug therapy and immunotherapy may be more likely to contribute to the treatment outcome and prognosis of patients. We will further investigate the possibility of different drug treatments for tumor subtypes.

### Research of somatic mutation and TMB

**FIGURE 10:**
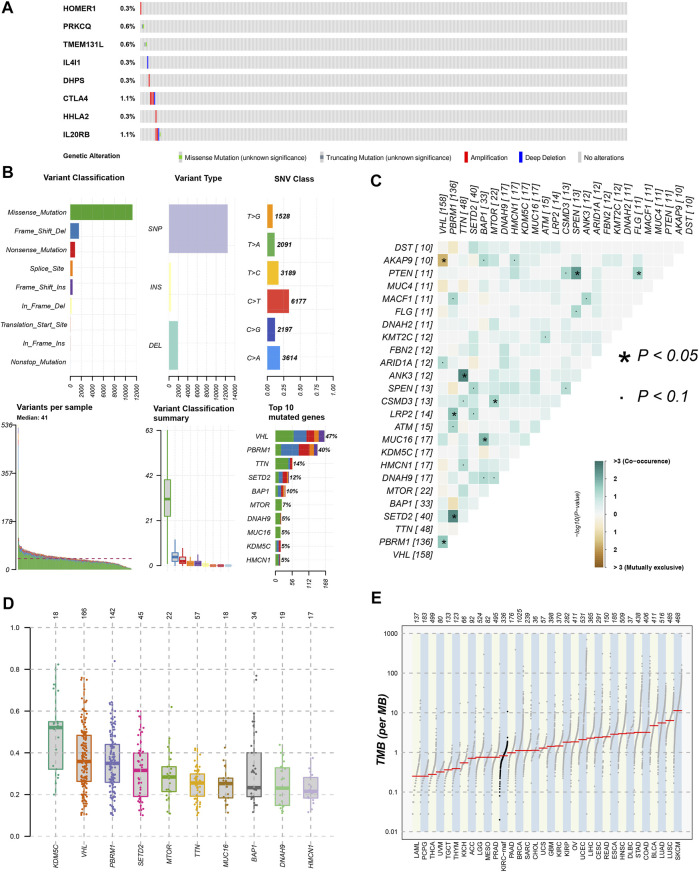
Gene mutation analysis of ccRCC patients. **(A)**The mutation of 8 modeling TRGs obtained from the cBioPortal database; **(B)** the mutation landscape of ccRCC patients in the TCGA database; **(C)** genes with mutually exclusive mutation or simultaneous mutation; **(D)** the Variant Allele Frequencies (VAF) boxplot of mutated genes; **(E)** the comparison of TMB among ccRCC and other tumors in TCGA database.

**FIGURE 11:**
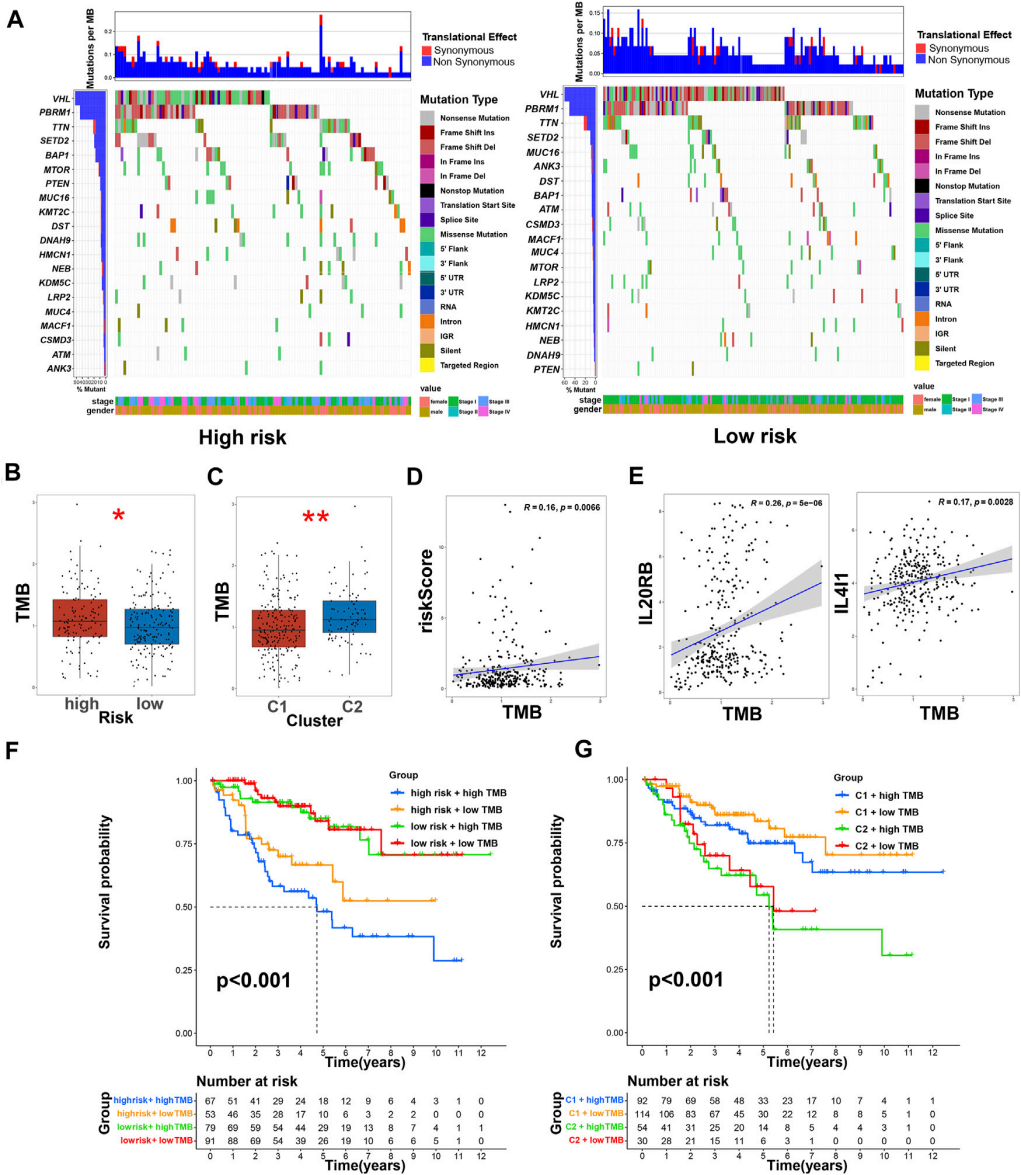
TMB and immunotherapy prediction of ccRCC patients. **(A)** The comparison of mutations of differentially expressed TRGs in high- and low-risk groups; **(B)** the comparison of TMB between low- and high-risk groups, * means *p* < 0.05; **(C)** the comparison of TMB between clusters, ** means *p* < 0.01; **(D)** the correlation of risk score and TMB score; **(E)** the correlation of TRGs and TMB scores; **(F)** Kaplan–Meier survival curves of OS of patients among different groups based on risk scores and TMB scores; **(G)** Kaplan–Meier survival curves of OS of patients among different groups based on clusters and TMB scores.

We used the cBioPortal database to analyze the mutations of eight TRGs involved in the modeling. However, we found that there were no significant mutations in these eight genes (Figure 10A). Thus, we analyzed all somatic mutations and visualized the information. The missense mutation was the most common variant classification and *VHL* is the gene with the highest mutation rate (Figure 10B). *VHL* and *AKAP9* are mutually exclusive mutants, while *VHL* and *PBRM1* are often co-mutated (Figure 10C). The variant allele frequencies (VAF) were mostly at a low level (Figure 10D). Compared with other tumors, the TMB of ccRCC was lower than the moderate level (Figure 10E). Besides, we studied all the differentially expressed TRGs and compared their mutations in high- and low-risk groups (Figure 11A). The results showed that the first four mutant genes, VHL, PBRM1, TTN, and SETD2, were the same between these two groups. The result was consistent with the report of previous studies (Braun, et al., 2020; Kim, et al., 2021). As a tumor suppressor gene, the mutation of Von Hippel-Lindau (VHL) interferes with the normal development and function of Follicular helper T (Tfh) cells by affecting glycolysis through the VHL-HIF-1 *α* axis (Zhu, et al., 2019). As for VHL-deficient T cells, the normal differentiation of Th17 cells was impaired *in vitro* (Chitrakar, et al., 2020). We calculated the TMB scores of ccRCC patients and compared them between high- and low-risk groups and between different clusters (Supplementary Table S9). The results suggested that patients with high-risk scores and patients belonging to Cluster2 have higher TMB scores (Figures 11B,C). Besides, the TMB score was positively correlated with the risk score (Figure 11D). Among the 8 TRGs participating in modeling, only the expression of IL4I1 and IL20RB was positively correlated with TMB scores (Figure 11E). In addition, we also analyzed the prognosis of the patients. Interestingly, patients with high-risk scores and high TMB scores had the worst prognosis. Similarly, patients belonging to Cluster2 with high TMB scores had the worst prognosis (Figures 11F,G).

## Discussion

As we all know, immune cells, especially T cells, play an irreplaceable role in the occurrence and development of tumors. For ccRCC, modern medical treatments such as targeted therapy and immunotherapy are carried out around immune cells. The importance of TRGs in anti-tumor is self-evident because of the function of regulating immune cell proliferation. In this study, we established a prognostic signature based on the TRGs found so far.

Our stratified model consists of eight TRGs (*CTLA4, IL4I1, HHLA2, PRKCQ, IL20RB, HOMER1, DHPS,* and *TMEM131L*). There were significant differences in prognosis and functional enrichment among the patients who were divided into high- and low-risk groups based on the stratified model. The prediction of immune cell infiltration shows that the TME of high-risk patients may enrich more Tregs and CD8+cells, which have been proved to inhibit tumor immune response in previous studies, thus helping tumor cells escape immune monitoring (Shang, et al., 2015; Tanaka and Sakaguchi, 2017; Dai, et al., 2021; Gao, et al., 2022). Of note, Tregs have the function of regulating T cells, B cells, NK cells, dendritic cells (DCs), and macrophages. It can deprive costimulatory signals of responder T cells by expressing CTLA4 and depriving the surrounding IL2. Importantly, Tregs also produce immunosuppressive cytokines such as TGF-β and IL10 which can inhibit the function of DCs and CD8^+^ effector T cells (Teffs) and promote the transformation of CD4+T cells into Tregs. Higher FOXP3+Tregs infiltration was found to be significantly associated with shorter OS in renal cell carcinoma (Shang, et al., 2015; Tanaka and Sakaguchi, 2017; Gao, et al., 2022). The decrease of Tregs can inhibit the growth of tumors and improve the effectiveness of tumor immunotherapy (Martin, et al., 2010). A higher Teff/Treg ratio in ccRCC was associated with a lower postoperative recurrence rate (Ghatalia, et al., 2019). Besides, Siyuan Dai et al. reported that excessive infiltration of CXCL13+CD8+T cells in tumors of ccRCC patients impaired the immune function of total CD8+T cells, which was associated with poor prognosis (Dai, et al., 2021). According to previous research, targeted therapy can often bring some adverse reactions and the therapeutic effects were different among individuals. Sometimes patients needed help with immunotherapy. However, tumors with different immune microenvironments had different sensitivities to immunotherapy. Highly invasive tumors with high immune scores were generally considered hot tumors, while non-invasive tumors with low immune scores were considered cold tumors. The distinction between hot and cold tumors can provide a reference for individualized immunotherapy based on tumor subtype clustering (Galon and Bruni, 2019; Kim, et al., 2021). Based on risk stratification, we couldn’t distinguish the difference in immune microenvironment between these two groups well. Therefore, we re-group patients with ccRCC according to tumor subtypes based on risk scores. After clustering, it can be seen that there were significant differences in the scores of immune microenvironments. Cluster2 has a higher stromal score, immune score, and estimate score than Cluster1. For hot tumors of Cluster2, we can use T-cell-targeting immunotherapies or other methods to treat patients. However, cold tumors often have a low mutation burden and rare invasive immune effector cells, which are resistant to a variety of immune checkpoint blocking drugs. We need to find ways to transform cold tumors into hot tumors. For instance, activating innate immune sensing pathways related to cancer is a potential method (Duan, et al., 2020; Liu, et al., 2020).

It is a pity that there are few immunotherapy data on ccRCC patients in the TCGA database. According to the latest research, TMB was significantly related to the efficacy of immunotherapy in tumor patients. There is increasing evidence that TMB is expected to become a predictive biomarker for immunotherapy of solid tumors such as lung cancer (Klein, et al., 2021; Vega, et al., 2021; Kim, et al., 2022). Therefore, we used TMB scores instead of immunotherapy data to validate our stratified model and hot and cold tumor subtypes. The results showed that there were significant differences in TMB scores between high- and low-risk groups and between tumor subtypes. We validated the stratified model internally through the test group and all samples. But it is difficult to verify the prognosis externally because there are few data containing both gene expression and survival data of ccRCC patients in external databases such as the Gene Expression Omnibus database. We used multiple platforms to analyze the immune microenvironment, which may be regarded as external verification in a sense. Our results have some limitations. More experiments are needed to verify and explore the possibility of new-found TRGs as new targets for immunotherapy in the future. We believed that our model is reasonable and can be verified by future clinical data and basic trials.

## Data Availability

Publicly available datasets were analyzed in this study. This data can be found here: 1) The Cancer Genome Atlas (TCGA) database. http://tcga.cancer.gov/. Accessed 29 March 2022. 2) University of California Santa Cruz Xena (UCSC Xena). https://xena.ucsc.edu/. Accessed 29 March 2022. 3) ImmPort database. https://www.immport.org/. Accessed 29 March 2022. 4) AmiGO2 database. http://amigo.geneontology.org/amigo/. Accessed 29 March 2022. 5) STRING database. https://www.string-db.org/. Accessed 29 March 2022. 6) cBioPortal database. https://www.cbioportal.org/. Accessed 29 March 2022.

## Glossary

AHR: Aryl hydrocarbon receptor
AUC: area under the ROC curve
ccRCC: clear cell renal cell carcinoma
CD4/8/28/80/86: CD4/8/28/80/86 molecule
chRCC: chromophobe renal cell carcinoma
CI: confidence interval
CTLA4: cytotoxic T lymphocyte antigen 4
DC: dendritic cell
DEG: differentially expressed gene
DHPS: Deoxyhypusine synthase
FDR: false discovery rate
FOXP3: Forkhead box P3
GO: Gene Ontology
GSEA: Gene Set Enrichment Analysis
HHLA2: HERV-H LTR-associating 2
HOMER1: Homer scaffold protein 1
HR: hazard ratio
IC50: half-maximal inhibitory concentration
ICB: immune checkpoint blocking
ICI: immune checkpoint inhibitor
IL2/4/6/10: Interleukin 2/4/6/10
IL20RB: Interleukin 20 receptor subunit beta
IL4I1: Interleukin 4 induced 1
KEGG: Kyoto Encyclopedia of Genes and Genomes
LASSO: least absolute shrinkage and selection operator
NES: normalized enrichment score
OS: overall survival
PCA: principal component analysis
PD1: programmed cell death protein 1
PPI: protein-protein interaction
pRCC: papillary renal cell carcinoma
PRKCQ: protein kinase C theta
ROC: receiver operating characteristic
ssGSEA: single-sample gene set enrichment analysis
TCGA: The Cancer Genome Atlas
Teff: effector T cell
Tfh: follicular helper T
TIL: tumor-infiltrating lymphocyte
TMB: tumor mutation burden
TME: tumor microenvironment
TMEM131L: Transmembrane 131 like
Treg: regulatory T cell
TRG: T cell proliferation-related gene
t-SNE: t-distributed stochastic neighbor embedding
UCSC Xena: University of California Santa Cruz Xena
VHL: Von Hippel-Lindau
