# Supplementary figures and images for: T cell proliferation-related genes: Predicting prognosis, identifying the cold and hot tumors, and guiding treatment in clear cell renal cell carcinoma
Source: Front Genet. 2022 Sep 2;13:948734. doi: 10.3389/fgene.2022.948734 (PMC9478955; doi:10.3389/fgene.2022.948734)

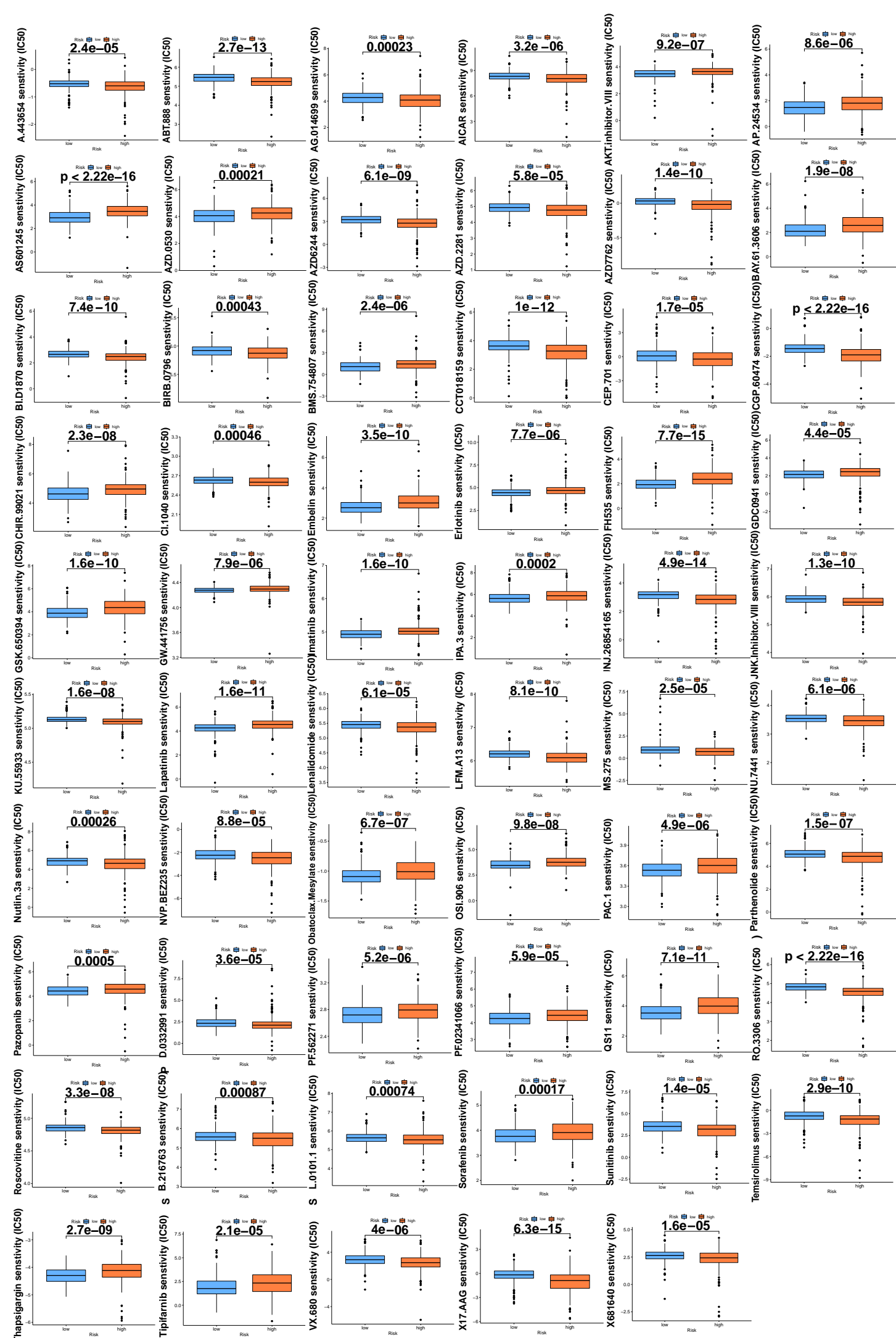

Supplementary Figure 2. All the sensitivities of targeted agents in high- and low-risk groups.

Supplement: Supplementary file 4 [file Image2.pdf]

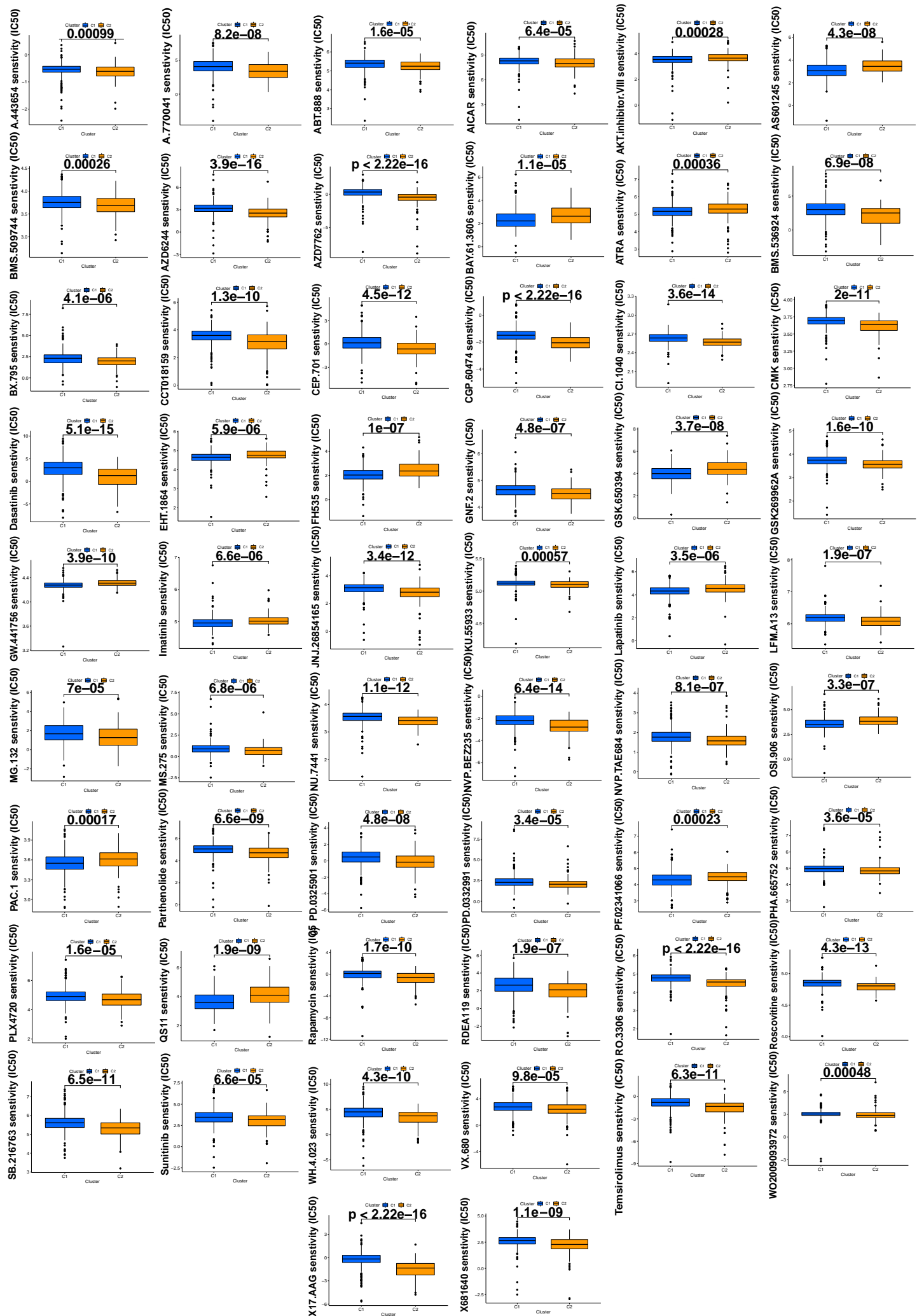

Supplementary Figure 3. All the sensitivities of targeted agents in Cluster1 and Cluster2.

Supplement: Supplementary file 6 [file Image3.pdf]
