# Supplementary material for: T cell proliferation-related genes: Predicting prognosis, identifying the cold and hot tumors, and guiding treatment in clear cell renal cell carcinoma
Source: Front Genet. 2022 Sep 2;13:948734. doi: 10.3389/fgene.2022.948734 (PMC9478955; doi:10.3389/fgene.2022.948734)
Supplement: Supplementary file 12 [file Image1.pdf]

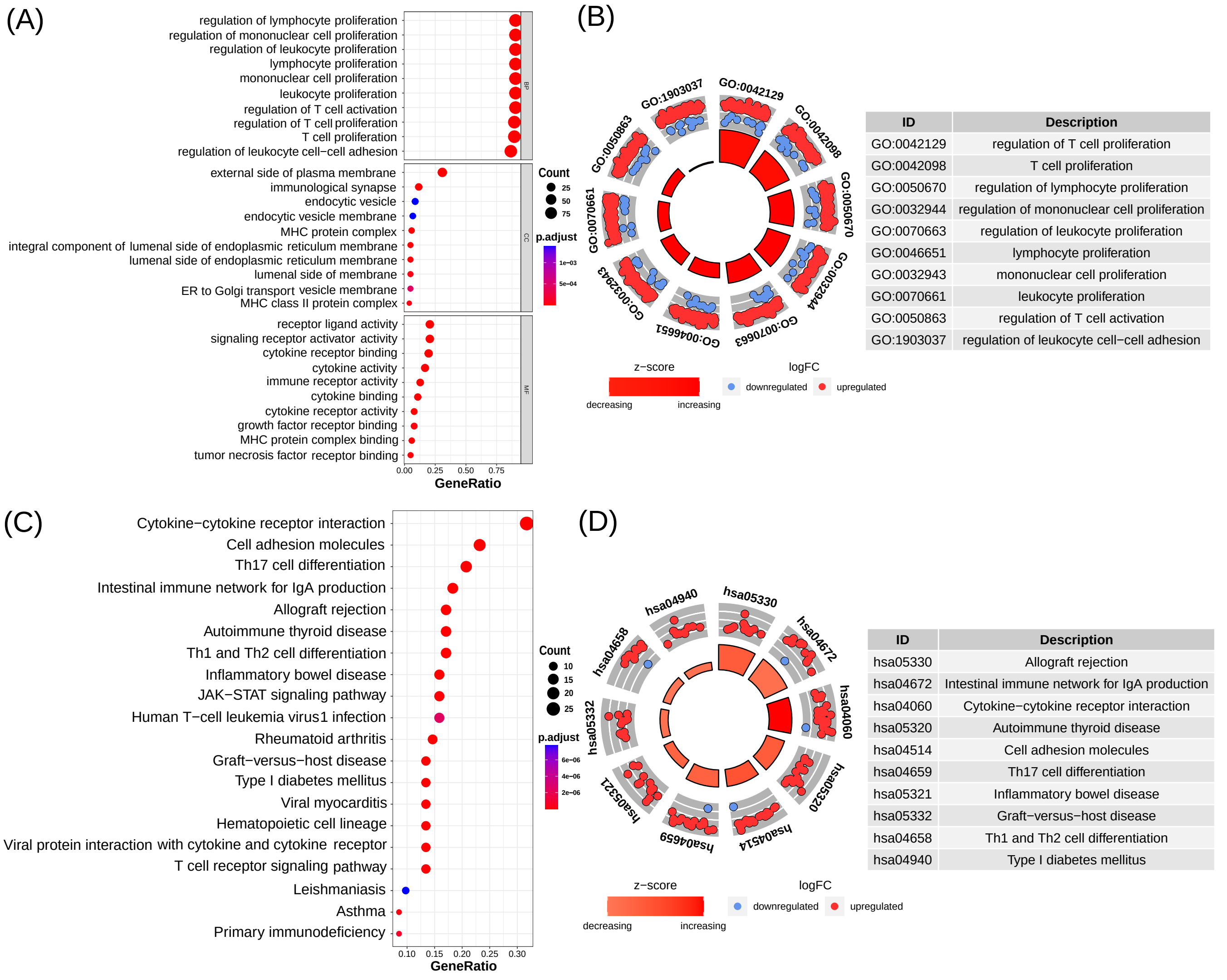

Supplementary Figure 1. Function and pathway enrichment of the differentially expressed TRGs. (A) The significant terms of GO function enrichment of the differentially expressed TRGs; (B) the GO circle showsthe scatter map of the Log2(fold change) of the differentially expressed TRGs; (C) the top 20 significantterms of KEGG analysis of the differentially expressed TRGs; (D) the KEGG circle shows the scatter mapof the Log2(fold change) of the differentially expressed TRGs.
